# Burden and Patterns of Oral Diseases and Systemic Comorbidities in Older Adults Attending Primary Care: A Sex- and Age-Stratified Analysis

**DOI:** 10.3390/medicina62071325

**Published:** 2026-07-09

**Authors:** Daniel Lopez-Hernandez, Osvaldo Erik Sanchez-Hernandez, Tabata Gabriela Anguiano-Velazquez, Leticia Brito-Aranda, Aline Vanessa Carrera-Vite, Aleli Julieta Izquierdo-Vega, Perla Veronica Salinas-Palacios, Josefina Reynoso-Vazquez, Abraham Espinoza-Perdomo, Nadia Esmeralda Crisantos-Reyes, Christian David Sevilla-Mendoza, Miriam Azucena Gonzalez-Sandoval, Marcos Meneses-Mayo, Arturo Salazar-Campos

**Affiliations:** 1 Clínica de Medicina Familiar “División del Norte”, Instituto de Seguridad y Servicios Sociales de los Trabajadores del Estado, Mexico City 04840, Mexico; 2 Area Academica de Medicina, Instituto de Ciencias de la Salud, Universidad Autonoma del Estado de Hidalgo, San Agustín Tlaxiaca 42090, Mexico; aleli_izquierdo11168@uaeh.edu.mx (A.J.I.-V.); arturo_salazar10347@uaeh.edu.mx (A.S.-C.); 3 Unidad de Medicina Familiar No. 03, Órgano de Operación Administrativa Desconcentrada Distrito Federal 2 Norte, Instituto Mexicano del Seguro Social, Mexico City 06600, Mexico; thathis7@hotmail.com (T.G.A.-V.); chrisevillamendo@gmail.com (C.D.S.-M.); 4 Centro de Investigación y de Educación Continua Sociedad Civil, Ciudad Nezahualcóyotl 57820, Mexico; letty.brito@gmail.com; 5 Clínica de Medicina Familiar “Guerrero”, Instituto de Seguridad y Servicios Sociales de los Trabajadores del Estado, Mexico City 06300, Mexico; zihanya@hotmail.com; 6 Centro Médico Nacional “20 de Noviembre”, Instituto de Seguridad y Servicios Sociales de los Trabajadores del Estado, Mexico City 03104, Mexico; perla.salinas@issste.gob.mx; 7 Area Academica de Farmacia, Instituto de Ciencias de la Salud, Universidad Autonoma del Estado de Hidalgo, San Agustín Tlaxiaca 42090, Mexico; jreynoso@uaeh.edu.mx; 8 Clínica de Medicina Familiar “Ermita”, Instituto de Seguridad y Servicios Sociales de los Trabajadores del Estado, Mexico City 09180, Mexico; abrahamep1@gmail.com; 9 Unidad de Medicina Familiar No. 35, Órgano de Operación Administrativa Desconcentrada Distrito Federal Norte, Instituto Mexicano del Seguro Social, Mexico City 08100, Mexico; nadiacrisrey5279@gmail.com; 10 Hospital General de Zona No. 27, Órgano de Operación Administrativa Desconcentrada Distrito Federal 2 Norte, Instituto Mexicano del Seguro Social, Mexico City 06900, Mexico; dra.miriam.gonzalez.anestesiologa@gmail.com; 11 Facultad de Ciencias de la Salud, Centro de Investigación en Ciencias de la Salud (CICSA), Universidad Anahúac México, Huixquilucan 52786, Mexico; marcos.meneses@anahuac.mx

**Keywords:** multimorbidity, non-communicable diseases, older adults, oral health

## Abstract

*Background and Objectives*: Oral health is a key but often overlooked component of healthy aging and functional wellbeing in older adults. Population aging, multimorbidity, and social determinants of health interact in order to shape oral disease patterns, particularly in primary care settings. Understanding age- and sex-specific distributions of oral diseases is essential for informing integrated public health strategies aimed at promoting healthy aging. *Materials and Methods*: We conducted a single-center, clinic-based cross-sectional study using the SIMEF primary care database, including 7386 adults aged 60 years and older, who were attended between January and December 2022. Oral diseases were identified using ICD-10 codes K00–K14. Prevalence estimates were calculated by life decade and sex. Associations were assessed using chi-square tests and sex-stratified analyses. *Results*: The overall prevalence of oral diseases was 5.2%, with a significant and progressive decline across age groups, from 6.5% in sexagenarians to 1.6% in nonagenarians (*p* < 0.001). The most prevalent conditions were disorders of teeth and supporting structures (2.6%, 95% confidence interval [95% CI] 2.3–3.0), dentofacial anomalies (2.2%, 95% CI 1.8–2.5), and dental caries (1.7%, 95% CI 1.4–2.0). Women showed a slightly higher prevalence of selected oral and mucosal conditions. Among older adults with oral diseases (*n* = 384), the most frequent comorbidities were hypertension (40.4% 95% CI 35.7–45.3), type 2 diabetes (29.2% 95% CI 24.7–33.8), and dyslipidemia (21.1% 95% CI 17.2–25.3), with marked sex-specific differences in endocrine, urinary, musculoskeletal, and mental health conditions. *Conclusions*: Oral disease burden in older adults decreases with advancing age but remains strongly linked to cardiometabolic and systemic comorbidities, reflecting shared risk factors relevant to healthy aging. The observed age- and sex-specific patterns underscore the need to integrate oral health into primary care and public health policies targeting aging populations, supporting comprehensive and equitable strategies for healthy longevity.

## 1. Introduction

Population aging represents a major public health challenge worldwide. By 2050, people aged 65 and above will surpass children under 5 years old worldwide [1], leading to an increased burden of non-communicable diseases and greater demands on health systems. According to the World Health Organization (WHO), by 2030, one in six people worldwide will be aged 60 years old or older [2], and their percentage globally by the year 2050 will nearly double from 12% to 22% [1,2]. Similarly, the population aged 80 years old and over is projected to triple between decades 2020 and 2050, reaching an estimated 426 million individuals worldwide [2]. This demographic transition first emerged in high-income countries [2]. However, it has accelerated in low- and middle-income countries [2]. It is estimated that by the year 2050, nearly two-thirds of the global population aged 60 years old and over will reside in these settings [2].

The implications of population aging pose significant challenges for health systems and societies at large, requiring substantial structural and policy adaptations [1,2,3]. Central to this transformation is the question of how individuals can maintain health and functional capacity across an extended lifespan [2]. As longevity increases, the emphasis must shift to living well for as long as possible.

In addition, this demographic shift is also accompanied by a growing prevalence of complex health conditions, referred to as geriatric syndromes [2]. Among the most prominent are frailty and sarcopenia, both characterized by reduced functional capacity and increased vulnerability to disease [2]. Frailty is associated with a range of adverse health outcomes, including mortality, falls, fractures, and institutionalization [2,3]. Moreover, older individuals frequently present with multiple comorbidities such as hypertension, diabetes, dyslipidemia, gonarthrosis, depressive episodes, risk of malnutrition, oral diseases, polypharmacy, frailty, and other geriatric syndromes, all of which pose significant challenges to conventional clinical management [4,5,6,7,8,9,10,11].

Oral diseases are highly prevalent and tend to accumulate over the life course, reflecting prolonged exposure to biological, behavioral, and social determinants. Among older adults, oral health is intrinsically linked to key functions such as mastication, nutritional intake, communication, and social engagement. Consequently, its deterioration may contribute to frailty, diminished life quality, and loss of autonomy and functional ability. Furthermore, oral health is not an isolated domain but is closely intertwined with systemic conditions—particularly cardiometabolic diseases [4]—through shared risk factors and underlying inflammatory mechanisms [5].

On the other hand, the health status of older populations is increasingly characterized by multimorbidity, declining physiological reserve, and complex care requirements. Within this framework, oral health shapes and is shaped by broader dimensions of wellbeing, including cognitive function, physical performance, and social support networks. Functional limitations, cognitive decline, and social disadvantage may hinder the maintenance of adequate oral hygiene and act as barriers to accessing dental care, resulting in unmet needs and disease progression. Additionally, compromised oral health may further aggravate systemic conditions, contributing to nutritional insufficiencies, and accelerating overall functional decline.

Consequently, promoting healthy and successful aging becomes a global priority. Indeed, population aging can be understood as a reflection of the same population health: societies that age are those that have achieved longer life expectancy. Therefore, ensuring that these years are lived in good health is essential for both individual wellbeing and sustainable social development. Oral health is a critical yet often overlooked component of overall wellbeing in older adults, with significant implications for nutrition, psychosocial health, and life quality. Therefore, maintaining health (including oral health) and functional ability in later life has become a central priority for public health and primary care.

In Mexico, evidence from populations receiving care through the Instituto de Seguridad y Servicios Sociales de los Trabajadores del Estado (ISSSTE, by its acronym in Spanish) has highlighted the high burden of oral diseases among older adults [12]. Previous studies have reported that dental caries, periodontal disease, tooth loss, and oral mucosal lesions are among the most frequent diagnoses, often coexisting with systemic comorbidities such as diabetes, hypertension, obesity, hypothyroidism, low back pain, gonarthrosis, and dyslipidemia [5,12,13]. These findings underscore the relevance of oral health within primary care, where many older adults receive ongoing management for chronic conditions but may have limited access to preventive dental services. Despite these prior insights, data on the prevalence of oral diseases and associated comorbidities in Mexican older adults remain limited. The primary objective of this study was to describe the epidemiological profile of oral diseases classified within the International Classification of Diseases, Tenth Revision (ICD-10) block K00–K14 among older adults receiving care at the primary healthcare level. Secondary objectives were (i) to evaluate differences in the distribution of oral diseases according to sex and age group and (ii) to characterize associated comorbidities using the ICD-10 classification system.

## 2. Materials and Methods

### 2.1. Study Design and Data Collection

A previously published secondary database was analyzed using a single-center, clinic-based cross-sectional design [13] . The study population consisted of patients receiving outpatient care at the Family Medicine Clinic (FMC) “División del Norte”, ISSSTE, in Mexico City, Mexico [13] . All the included participants were identified through the Medical Financial Information System (SIMEF, Mexico City, Mexico–System), the institutional electronic system routinely used to record outpatient consultations at this healthcare unit [13] The dataset comprised medical records of older adult patients attending primary healthcare services between January and December 2022 [13] . Overall, the original database contained 73,974 medical records corresponding to 17,918 individuals spanning all age groups [13] . For this study, only medical records corresponding to individuals aged 60 years and older were considered eligible. The study was conducted from 1 June 2025 to 28 February 2026.

#### The International Classification of Diseases 10th Revision Codes Validation

The ICD-10 provides an internationally standardized framework for recording, coding, and classifying diseases and health conditions across healthcare systems. In this study, ICD-10 diagnoses were assigned according to institutional coding procedures in which healthcare professionals systematically documented clinical conditions during routine medical care, and these were subsequently subjected to systematic verification by trained statistical personnel to minimize potential misclassification. This process enhances coding accuracy and diagnostic consistency, thereby minimizing variability attributable to individual clinical judgement.

Only records containing complete diagnostic data were incorporated into the analytical database in order to strengthen the consistency and dependability of the coded information. Although administrative health databases may present unavoidable limitations, the application of standardized ICD-10 definitions, together with trained personnel and regular quality-control procedures, provided a reliable methodological basis for the accurate identification of the diseases which were evaluated in this study.

### 2.2. Patient Selection and Study Population

The preparation of the study database involved several sequential procedures. Initially, monthly reports corresponding to the study period were extracted from the SIMEF system in Excel format. Subsequently, all files were then merged into a unified dataset and subjected to a data cleaning process to identify inconsistencies, duplicates, and incomplete records.

The patient was the unit of analysis. Initial patient identification was based on full name, age, sex, and file number. Because patients could have multiple outpatient visits during the study period, diagnostic information was consolidated at the patient level. For each condition, a patient was classified as having the disease if the corresponding ICD-10 diagnostic code was recorded at least once during any consultation within the study year. This procedure prevented duplication of diagnoses arising from repeated visits. A unique anonymized study identifier was then generated for each individual to allow for the deterministic linkage of all variables corresponding to the same patient and to prevent duplication.

After cleaning the database integration, a quality-control process was performed in order to assess completeness and internal consistency. Records were subsequently screened according to eligibility criteria, and individuals who did not meet the requirements were excluded. A census of eligible older adults (≥60 years old) was then constructed. The final dataset was used for statistical analysis.

The eligibility criteria are as follows:

1. Records were considered eligible when they corresponded to adults aged 60 years old or older, regardless of sex, and when at least one outpatient consultation had been registered in the SIMEF database during the established study period. In addition, only records containing complete demographic and clinical information—including patient identification variables (name, file number), beneficiary type, consultation dates, and ICD-10 diagnostic codes—were incorporated into the analysis.

The exclusion criteria are as follows:

1. Records were excluded when they belonged to individuals younger than 60 years of age or when the available information was insufficient for analysis due to missing, inconsistent, or inaccurate demographic or diagnostic data within the SIMEF system. Likewise, duplicate entries detected during the database depuration process were removed prior to statistical evaluation.

### 2.3. Sampling and Data Procedure

A population-based census approach was applied by incorporating every record that fulfilled the established selection criteria from the final consolidated database. This strategy allowed for the inclusion of only valid records with sufficient completeness and internal coherence for analysis.

The institutional database comprised 17,918 subjects of all ages and contained 2491 ICD-10 diagnostic records (Supplementary Figure S1 and Supplementary Table S1). From this broader population, 7386 adults aged 60 years and older met the eligibility criterion and constituted the study population. Within this older adult cohort, 1637 unique four-character ICD-10 subcategories were documented and used to characterize the diagnostic profile of the population (Supplementary Figure S1).

Among these older adults, 384 patients had at least one diagnosis classified within the ICD-10 block K00–K14 (diseases of oral cavity, salivary glands and jaws), whereas 7002 had no diagnosis within this block (Supplementary Figure S1). Within the oral disease cohort, 492 unique four-character ICD-10 subcategories distributed across multiple ICD-10 chapters were identified and subsequently used to characterize oral diseases and associated comorbidities.

According to the ICD-10 hierarchical classification, 108 of these four-character subcategories were classified within Chapter XI (diseases of the digestive system; K00–K93). Within Chapter XI, 66 four-character subcategories belonged to the K00–K14 block (diseases of oral cavity, salivary glands and jaws), encompassing all 15 three-character oral disease categories (K00–K14). These categories constituted the basis for the identification and classification of oral diseases in the study cohort. The hierarchical process used for population selection and ICD-10 classification is illustrated in Supplementary Figure S1.

Additional analytical variables were constructed to classify cases of obesity, dyslipidemia, and oral diseases. Obesity was identified through ICD-10 category E66. For the obesity variable, the diagnostic four-character subcategories E66.0; E66.8; and E66.9 were included. Dyslipidemia was defined using ICD-10 category E78, selecting the four-character subcategories E78.0; E78.1; E78.2; and E78.5. For oral diseases, disorders of the oral cavity, salivary glands, and jaws (ICD-10 block K00–K14) were included. This group comprises three-character categories related to abnormalities in tooth development and eruption (K00), impacted and/or embedded teeth (K01), dental caries (K02), other diseases of dental hard tissues (K03), pulp and periapical disorders (K04), gingival and periodontal diseases (K05), disorders affecting the gingiva and edentulous alveolar ridge (K06), dentofacial abnormalities (including malocclusion) (K07), disorders involving teeth and supporting structures (K08), cystic lesions of the oral region (K09), jaw diseases (K10), salivary gland diseases (K11), stomatitis and associated lesions (K12), diseases affecting the lip and oral mucosa (K13), and tongue diseases (K14). The complete distribution of diagnostic categories is presented in Supplementary Table S2.

To estimate the prevalence of selected comorbidities, all ICD-10 codes were operationalized as dichotomous variables, where 0 indicated absence and 1 indicated presence of the condition. Each condition was analyzed as an independent comorbidity. In addition, oral and dental diseases were assessed both as a composite outcome (all categories K00–K14 combined) and as disaggregated categories according to ICD-10 (K00–K14). Prevalence estimates were calculated for the overall study population. Sex-specific prevalence was also estimated and further stratified by decade of life, enabling a detailed characterization of age- and sex-specific patterns. This analytical strategy allowed for the estimation of disease prevalence and the characterization of comorbidity profiles within the study population while maintaining methodological uniformity across the analyzed data.

### 2.4. Variables and Statistical Analysis

Only datasets with complete information were retained for statistical evaluation. The study variables included patient age, biological sex, and associated clinical conditions identified through ICD-10 diagnostic classifications. Overall, 492 different ICD-10 diagnostic entries were examined.

A descriptive analysis of qualitative variables was expressed as frequencies and proportions. In addition, continuous variables were summarized using measures of central tendency and dispersion, including mean values, standard deviation (SD), and interquartile range (IQR). Confidence intervals at the 95% level were calculated when relevant.

Associations between qualitative variables were assessed using the chi-square test with Yates’s correction, likelihood ratio chi-square analysis, or Fisher’s exact test according to the characteristics of the data. Numerical variables were evaluated using the median test for independent samples. Statistical significance was established at a two-tailed *p* value lower than 0.05.

### 2.5. Analysis Strategy

The statistical evaluation was structured into two complementary phases according to the aims of the investigation. Initially, a general descriptive assessment of the study cohort was carried out to identify the demographic characteristics and clinical features of older adults presenting oral diseases. Subsequently, the analysis was stratified by sex and by decades of life—defined as sexagenarians (60–69 years old), septuagenarians (70–79 years old), octogenarians (80–89 years old), nonagenarians (90–99 years old), and centenarians (≥100 years old)—to explore demographic profiles across age groups. In the second stage, bivariate comparisons were carried out to examine differences by sex and age in demographic variables, clinical features, and comorbidities.

### 2.6. Ethical Considerations

The study protocol was approved by both the Research Committee and the Research Ethics Committee of the FMC “División del Norte” (approval No. MFDN/SM//EZ/315/2024; 6 February 2024). The investigation was conducted in accordance with the ethical principles of the Declaration of Helsinki and national Good Clinical Practice regulations. Before statistical analysis, all records were anonymized by replacing personal identifiers with unique alphanumeric codes, thereby ensuring participant traceability without revealing individual identities. Access to identifiable information was restricted exclusively to the principal investigators responsible for data management. Once the analyses had been completed, only statistical results were shared with the remaining members of the research group, thereby safeguarding participant confidentiality throughout all stages of the investigation [13] .

## 3. Results

### 3.1. Characteristics of the Study Population

**Figure 1:**
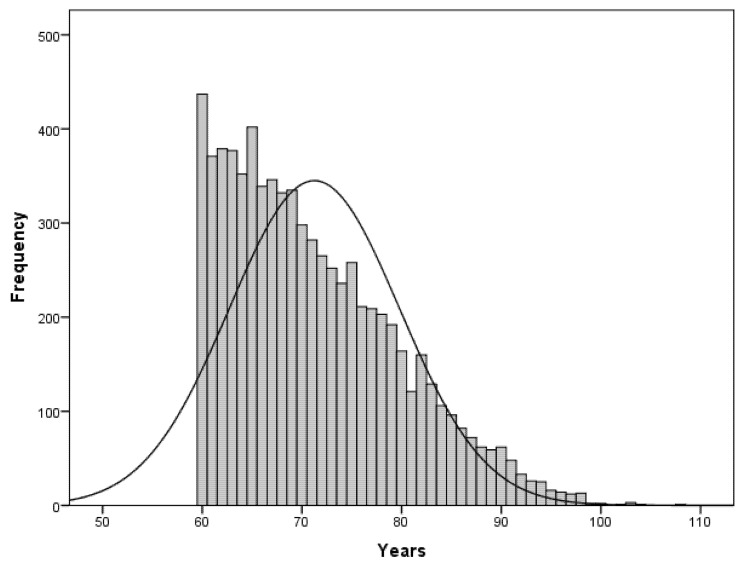
Source: Prepared by the authors using the results from the SIMEF database, January–December, 2022.

A total population of 7386 older adults were included (patients without oral diseases: 7002; 94.8%, 95% CI 94.3–95.3; oral disease patients: 384; 5.2%, 95 CI 4.7–5.7). Most of the participants are females (*n* = 4496; 60.9%, 95% CI 59.8–62.0 versus males *n* = 2890; 39.1%, 38.0–40.2). The age distribution of the elderly population showed an average age of 71.25 years old (SD = 8.54), with values ranging from 60 to 108 years old, indicating a broad age span across the sample (Figure 1). Likewise, the median age in the total population was 70 years old (IQR: 64–77 years). In addition, the median age was significantly higher among males (70 years old, IQR: 65–77) than among females (69 years old, IQR: 64–77) (independent median test, *p* = 0.031).

When stratified by age group, the distribution of participants showed a predominance of sexagenarians, with the proportion decreasing progressively as age increased. This pattern reflects the typical demographic trend in older populations, where the number of individuals declines in the more advanced age groups due to age-related morbidity and mortality (Table 1).

### 3.2. Prevalence of Oral and Dental Diseases in Elderly Population

Table 1 presents the overall prevalence of oral diseases, calculated considering all causes classified under ICD-10 codes into the block K00–K14, among the elderly population stratified by decade of life. The prevalence of oral and dental diseases declined progressively and significantly with age, from 6.5% in sexagenarians to 1.6% in nonagenarians, confirming a decrease in the burden of dental and oral disease in the oldest age groups. These findings suggest that the lower prevalence observed in advanced age might be related to extensive tooth loss and edentulism. As individuals retain fewer natural teeth, the opportunity to detect caries and periodontal disease may decrease, potentially contributing to the lower prevalence observed in this age group.

Table 2 presents the sex-stratified distribution of oral and dental diseases, classified according to the ICD-10 codes (K00–K14: diseases of oral cavity, salivary glands, and jaws), grouped by subchapter. The most prevalent categories were other disorders of teeth and supporting structures (K08, 2.6%), dentofacial anomalies [including malocclusion] (K07, 2.2%), and dental caries (K02, 1.7%). Women showed a slightly higher prevalence of other disorders of teeth and supporting structures and other diseases of lip and oral mucosa. These findings indicate a mild sexual dimorphism in the distribution of specific oral disease patterns among older adults.

Table 3 displays the prevalence of oral diseases by age group according to ICD-10 classification. Overall, dental caries, dentofacial anomalies [including malocclusion], and other disorders of teeth and supporting structures were the most common diagnoses in sexagenarians, with frequencies decreasing markedly in older age groups. Statistically significant age-related associations were found for embedded and impacted teeth, dental caries, other diseases of hard tissues of teeth, dentofacial anomalies, and other disorders of teeth and supporting structures (*p* < 0.05). The pattern indicates a reduction in both the frequency and diversity of oral pathologies with advancing age, consistent with age-related tooth loss and reduced exposure to etiological factors in late life.

### 3.3. Burden of Comorbidities of Adult Older Patients with Oral Diseases

Table 4 presents the distribution of the ten most frequent comorbidities among the elderly population diagnosed with oral and dental diseases (*n* = 384). The analysis was first conducted in the total population and subsequently stratified by sex. The most prevalent systemic conditions were hypertension, type 2 diabetes, and dyslipidemias, followed by obesity and chronic venous insufficiency. Females showed higher proportions of hypothyroidism, urinary tract infection, and anxiety disorder, while acute pharyngitis was slightly more common in males. Statistically significant sex-based differences were identified for hypothyroidism (*p* < 0.01), urinary tract infection (*p* < 0.01), anxiety disorder (*p* < 0.01), and low back pain (*p* = 0.042). These findings indicate that systemic comorbidities cluster differently between sexes in older adults with oral diseases, potentially reflecting biological, hormonal, and behavioral determinants underlying disease coexistence.

### 3.4. Sex-Specific Distribution of the Main Comorbidities in Older Adult Patients with Oral Diseases

Among males with dental diseases, the predominant comorbidities were hypertension (58 cases; 37.4%, CI 95% 29.8–45.0) and type 2 diabetes (43 cases; 27.7%, CI 95% 20.7–34.8). These conditions frequently coexist and share modifiable risk factors such as poor diet, obesity, and sedentary behavior, which are also associated with oral inflammatory diseases. Other common diagnoses included benign prostatic hyperplasia (31 cases; 20.0%, CI 95% 13.7–26.3), dyslipidemia (28 cases; 18.1%, CI 95% 12.0–24.1), and obesity (21 cases; 13.5%, CI 95% 8.2–18.9), highlighting the burden of chronic dysfunction. Acute pharyngitis (20 cases; 12.9%, CI 95% 7.6–18.2) was also reported, suggesting a component of recurrent upper respiratory infection, which is potentially related to oral microbiota imbalance.

Additionally, vascular and systemic conditions such as chronic venous insufficiency (13 cases; 8.4%, CI 95% 4.0–12.8), chronic ischemic heart disease (10 cases; 6.5%, CI 95% 2.6–10.3), sleep apnea (9 cases; 5.8%, CI 95% 2.1–9.5), gastro-esophageal reflux disease (9 cases; 5.8%, CI 95% 2.1–9.5), and prediabetes (8 cases; 5.2%, CI 95% 1.7–8.6) further illustrate the clustering of metabolic risk. Therefore, the male profile indicates a predominance of cardiometabolic comorbidity, consistent with systemic inflammation, dietary risk, and behavioral factors linked to both oral and general health deterioration.

In females, the most frequent comorbidities were hypertension (97 cases; 42.4%, 95% CI 36.0–48.8) and type 2 diabetes (69 cases; 30.1%, 95% CI 24.2–36.1), revealing a predominant cardiometabolic profile similar to that observed in males. Likewise, dyslipidemias (53 cases; 23.1%, 95% CI 17.7–28.6) and obesity (38 cases; 16.6%, 95% CI 11.8–21.4) were frequent, reinforcing the link between oral health and components of the metabolic syndrome. Other relevant chronic conditions included chronic venous insufficiency (33 cases; 14.4%, 95% CI 9.9–19.0), hypothyroidism (30 cases; 13.1%, 95% CI 8.7–17.5), and urinary tract infection (26 cases; 11.4%, 95% CI 7.2–15.5), with the latter two being more frequent among women and often associated with hormonal and anatomical factors.

Moreover, musculoskeletal and psychosomatic conditions such as low back pain (25 cases; 10.9%, 95% CI 6.9–15.0) and anxiety disorder (22 cases; 9.6%, 95% CI 5.8–13.4) were also common, highlighting the interplay between chronic pain, stress, and oral health. Additionally, acute pharyngitis (21 cases; 9.2%, 95% CI 5.4–12.9) appeared as a recurrent acute condition (20 cases; 12.9%, 95% CI 7.6–18.2). This female profile reflects a multidimensional burden of comorbidity, encompassing cardiometabolic, endocrine, musculoskeletal, and mental health disorders. Such complexity may influence both access to care and treatment outcomes in dental health, emphasizing the need for integrated care strategies that address the intersection of oral and systemic health, particularly in women with multiple chronic conditions.

## 4. Discussion

The epidemiological characterization of populations is essential to public health, as it enables the identification of disease patterns, the distribution of health needs, and the development of targeted and equitable interventions [14,15,16,17]. This is particularly relevant in aging societies, where the increasing proportion of older adults reflects the cumulative effects of biological, behavioral, and social exposures over time.

In the present study, oral diseases were identified in 5.2% of older adults, a prevalence lower than that reported in many population-based studies [18,19,20,21]. The lower prevalence of oral diseases observed in the oldest age groups may be partially explained by the high frequency of tooth loss and edentulism among older adults, which reduces the number of teeth susceptible to dental and periodontal conditions. This interpretation is biologically plausible but could not be directly evaluated because the information on tooth retention and edentulism was not available in the SIMEF database [22,23,24]. The progressive decline observed across age groups—from 6.5% in sexagenarians to 1.6% in nonagenarians—supports this hypothesis and is consistent with findings reported in other settings. Additionally, the relatively small number of individuals in the most advanced age groups may have contributed to the lower frequency of recorded conditions.

Several factors beyond biological aging have been proposed to influence oral health in older adults. Oral health in this populational group is strongly influenced by functional capacity, cognitive capacity, and social context. Some studies have shown that individuals with greater dependency in basic and instrumental activities of daily living are more likely to present poor oral hygiene, untreated dental conditions, and reduced access to dental care [25]. In addition, functional decline may reduce the likelihood of seeking preventive or curative services.

Similarly, cognitive impairment has been consistently associated with worse oral health outcomes [25,26,27,28,29,30,31]. Older adults with cognitive decline often experience difficulties in maintaining oral self-care, increasing the risk of oral infections and inflammatory conditions. In this setting, oral health may both influence and be affected by cognitive status, as chronic inflammation and poor nutritional intake related to oral dysfunction have been linked to accelerated cognitive decline [25,26,27,28,29,30,31].

Limited social support, social isolation, and exposure to elder abuse have been associated with poorer oral health, reduced utilization of dental services, and poorer overall health, partly through financial, structural, and behavioral barriers [32,33,34].

The findings of the present study also underscore a substantial burden of multimorbidity, particularly cardiometabolic conditions such as hypertension, type 2 diabetes, and dyslipidemia. These disorders frequently coexist with polypharmacy, which carries significant implications for oral health. The use of multiple medications has been consistently associated with xerostomia, reduced salivary flow, and a greater susceptibility to dental caries, periodontal disease, and oral infections [35,36]. This is especially relevant in older adults, in whom long-term pharmacological treatment further exacerbates oral vulnerability.

These observations align with growing evidence that oral and systemic health are closely interconnected [21]. Oral diseases and non-communicable diseases share common risk factors, including high sugar intake, tobacco use, and social determinants of health [21]. Periodontal disease has been strongly associated with cardiometabolic conditions such as diabetes and cardiovascular disease through shared inflammatory pathways [21]. This bidirectional relationship suggests that poor oral health may not only result from systemic disease but also contribute to its progression by amplifying systemic inflammation and complicating disease management.

In addition to clinical conditions, oral health plays a fundamental role in maintaining essential daily functions, including eating, speaking, and social interaction [21]. Among older adults, conditions such as tooth loss, hyposalivation, oral infections, and malignancies can significantly impair oral function, negatively affecting nutritional intake, psychosocial wellbeing, and overall life quality [21]. Indeed, edentulism—reported to affect a substantial proportion of the elderly population (78%)—has been linked to dietary modifications characterized by reduced consumption of fruits and vegetables and increased intake of energy-dense, soft foods rich in saturated fats and cholesterol [34]. Such dietary patterns may further aggravate cardiometabolic risk, thereby reinforcing a detrimental cycle between oral and systemic health.

Another potential mechanism proposed in the literature involves the oral microbiota, which has been suggested to contribute to systemic metabolic regulation, particularly through pathways related to insulin resistance [37,38]. Although most research has focused on the gut microbiota, alterations in microbial composition—characterized by shifts in bacterial phyla and increased inflammatory signaling—have been shown to influence metabolic pathways, intestinal permeability, and host immune responses [37,38]. These processes can lead to chronic low-grade inflammation, impaired insulin signaling, and increased cardiometabolic risk [37,38]. Given the anatomical and functional continuity between the oral cavity and the gastrointestinal tract, dysbiosis of the oral microbiome may similarly contribute to systemic inflammation and metabolic dysregulation. Moreover, the oral cavity may act as both a reservoir and a modulator of microbial communities that influence host metabolism. Periodontal pathogens and oral infections can promote systemic dissemination of inflammatory mediators and bacterial components, such as lipopolysaccharides, which activate immune pathways involved in insulin resistance [38]. This provides a plausible biological mechanism linking poor oral health with metabolic disorders, further supporting the need for integrated preventive and therapeutic approaches. Although causal relationships cannot be inferred from the present study, the observed patterns are consistent with previous evidence suggesting that oral health is closely interconnected with general health, particularly in populations with a high burden of chronic diseases. Addressing oral conditions in conjunction with systemic comorbidities may offer an opportunity to improve clinical outcomes, enhance quality of life, and reduce the overall burden of disease in aging populations.

Moreover, the decline in oral function has been associated with broader functional deterioration, frailty, sarcopenia, and eventual loss of independence in older individuals [25,26,27,28,29,30,39], supporting the inclusion of oral health as a fundamental component of healthy aging. Previous studies have even suggested that functional decline, often conceptualized as a progressive loss of intrinsic capacity or physical resilience, may further complicate this interaction [40,41,42]. Given the rapid growth of the aging population, there is an urgent need to develop comprehensive healthcare strategies that address both systemic and oral conditions in an integrated manner.

Sex differences observed in this study, including variations in comorbidity profiles and specific oral conditions, further support the need for tailored approaches. Women showed a higher prevalence of certain chronic and psychosocial conditions, such as hypothyroidism, urinary tract infections, and anxiety disorders, which may interact with oral health through hormonal, behavioral, and healthcare access pathways. These patterns underscore the importance of adopting a gender-sensitive perspective in both research and clinical practice.

Evidence from previous studies also suggests that barriers to accessing dental care may represent an important challenge among older adults with functional limitations, cognitive impairment, or limited social support. In addition, evidence from multiple settings indicates that these populations are less likely to receive regular dental care, leading to delayed diagnosis and treatment. This contributes to a cycle of deterioration, particularly in the presence of multimorbidity and dependency.

Therefore, it is essential to integrate oral health into comprehensive care models for older adults. Approaches that prioritize functional ability, interdisciplinary care, and continuity of services provide a valuable framework for addressing these complex and interrelated needs. Incorporating routine oral health assessments into primary care, alongside evaluation of functional, cognitive, and social domains, may improve early detection, optimize management, and enhance quality of life.

Finally, oral health in older adults may be viewed as one component of a broader health context that includes functional status, cognitive capacity, social support, and multimorbidity.

### Strengths and Limitations

This study has several limitations that should be considered when interpreting the findings. First, the analysis was based on ICD-10 diagnostic records extracted from the SIMEF system. Although oral diagnoses were established and recorded by qualified dental professionals as part of routine clinical practice, the use of secondary clinical records restricted the analysis to the variables available within the database, which did not permit independent verification of diagnostic procedures or clinical assessments. Second, the database did not contain information on the number of remaining teeth, edentulism status, denture use, disease severity, oral hygiene practices, or socioeconomic characteristics. Consequently, potential explanations for the lower frequency of oral diseases observed in the oldest age groups, including tooth loss and edentulism, should be regarded as plausible hypotheses rather than conclusions directly supported by the available data.

Third, the cross-sectional design precludes the establishment of temporal relationships or causal inferences between oral diseases and systemic comorbidities. Fourth, although comorbidities were described among patients with oral diseases, the absence of multivariable analyses prevented the assessment of independent epidemiological associations and also prevented the adjustment for potential confounding factors. Finally, this was a single-center clinic-based study conducted within the ISSSTE healthcare system. Therefore, caution is required when extrapolating the findings to other healthcare systems or community-based populations. Nevertheless, the results may provide useful evidence for primary care settings with organizational and demographic characteristics similar to those found in other regions of Mexico and Latin America.

Despite these limitations, the study has several important strengths. It included a census of all eligible older adults receiving care during the study period, thereby eliminating sampling procedures and providing a comprehensive characterization of oral disease diagnoses within the study population. The large sample size enabled age- and sex-stratified analyses across different stages of older adulthood. Furthermore, the use of a standardized ICD-10 framework allowed for the systematic classification of oral diseases and facilitated their evaluation within a primary healthcare context.

An additional strength and original contribution of this study lie in the use of routinely collected clinical information to characterize the distribution of ICD-10 oral disease categories among older adults receiving primary care services. Whereas most previous investigations have focused on specific oral conditions or relied exclusively on clinical examination protocols, the present study provides a comprehensive overview of oral disease categories and their coexistence with common chronic conditions in a real-world clinical setting. These findings contribute to a better understanding of oral health patterns among older adults and illustrate the potential value of health information systems for epidemiological surveillance, service planning, and the integration of oral health into primary care.

Moreover, it is important to acknowledge the relatively high frequency of certain diagnostic categories, which may be influenced by both true clinical demand and heterogeneity in diagnostic coding practices. To address this issue, the SIMEF database underwent a structured multi-stage validation process, including systematic data consolidation, internal consistency checks, and the removal of duplicate and incomplete records. Diagnostic entries were generated through institutional coding procedures performed by healthcare professionals during routine clinical care and subsequently subjected to standardized quality-control procedures by trained personnel, with the aim of improving coding accuracy and reducing inconsistencies. Nevertheless, as with all secondary healthcare databases, residual limitations related to completeness of recording, variability in diagnostic specificity, and potential under- or over-coding cannot be entirely excluded.

Importantly, despite these inherent limitations, the methodological approach applied in this study strengthens the robustness of the estimates. The use of a census-based design, incorporating all eligible records from the study period, together with the construction of a final analytical dataset at the individual patient level, minimized selection bias and avoided duplication of information arising from multiple consultations per patient. In addition, prevalence estimates were calculated exclusively at the patient level, ensuring that each individual contributed only once to the analysis. This approach reduces inflation bias commonly observed in consultation-based datasets and enhances the validity and interpretability of the epidemiological estimates. Therefore, although administrative data limitations must be considered, the methodological rigor applied in data processing and analysis supports the reliability of the findings.

## 5. Conclusions

This study highlights that the prevalence of oral diseases in older adults is relatively low and declines significantly with age. Oral conditions were concentrated in earlier stages of older age and were predominantly related to dental structures.

A substantial burden of multimorbidity was identified, particularly cardiometabolic conditions, reinforcing the shared risk factor framework between oral and systemic diseases. The presence of sex-specific differences further indicates the need for tailored clinical and public health approaches.

These findings underscore the limitations of conventional oral health indicators in aging populations and emphasize the need for more comprehensive measures that account for functional status and tooth retention.

On the other hand, from a public health perspective, integrating oral health into primary care and chronic disease management strategies is essential. Health systems should strengthen preventive, diagnostic, and rehabilitative services for older adults, incorporating oral health into routine geriatric assessment.

A life-course and person-centered approach is required to address the complex interaction between oral health, multimorbidity, and aging, with the aim of improving quality of life and reducing health system burden.

## Figures and Tables

**Table 1:** Prevalence of oral diseases—all causes—(ICD-10 K00–K14) in the elderly population.

	Sexagenarians *n*; % (95% CI)	Septuagenarians *n*; % (95% CI)	Octogenarians *n*; % (95% CI)	Nonagenarians *n*; % (95% CI)	Centenarians *n*; % (95% CI)	TP *n*; % (95% CI)
Without OD	3431; 93.5% (92.7–94.2)	2293; 95.3% (94.4–96.1)	1023; 97.3% (96.1–98.2)	247; 98.4% (96.1–99.3)	8; 100% (59.8–100.0)	7002; 94.8% (94.2–95.3)
OD	239; 6.5% (5.8–7.3)	113; 4.7% (3.9–5.6)	28; 2.7% (1.8–3.9)	4; 1.6% (0.5–4.1)	0; 0% (0.0–0.0)	384; 5.2% (4.7–5.8)
Total	3670	2406	1051	251	8	7386

Source: Prepared by the authors using the results from the SIMEF database, January–December, 2022. TP: total population. 95% CI: 95% confidence interval. *n* = 7386. OD = oral diseases. Sexagenarians (60–69 years old). Septuagenarians (70–79 years old). Octogenarians (80–89 years old). Nonagenarians (90–99 years old). Centenarians (≥100 years old). Comparisons between age group were performed using the likelihood ratio chi-square (likelihood ratio χ^2^ = 39.586, degree freedom = 4, *p* < 0.001).

**Table 2:** Prevalence and sex-based comparison of oral diseases (ICD-10: K00–K14) according to the International Classification of Diseases, 10th Revision (ICD-10).

ICD-10 Codes	Total Population *n*; % (95% CI)	Females *n*; % (95% CI)	Males *n*; % (95% CI)
K00	3 (0.0%, 0.0–0.1)	2 (0.0%, 0.0–0.1)	1 (0.0%, 0.0–0.1)
K01	14 (0.2%, 0.1–0.3)	6 (0.1%, 0.0–0.2)	8 (0.3%, 0.1–0.5)
K02	122 (1.7%, 1.4–2.0)	66 (1.5%, 1.1–1.8)	56 (1.9%, 1.5–2.5)
K03	48 (0.6%, 0.5–0.8)	27 (0.6%, 0.4–0.8)	21 (0.7%, 0.4–1.0)
K04	103 (1.4%, 1.1–1.7)	56 (1.2%, 0.9–1.6)	47 (1.6%, 1.1–2.1)
K05	65 (0.9%, 0.7–1.1)	45 (1.0%, 0.7–1.3)	20 (0.7%, 0.4–1.0)
K06	21 (0.3%, 0.2–0.4)	14 (0.3%, 0.2–0.5)	7 (0.2%, 0.1–0.4)
K07	159 (2.2%, 1.8–2.5)	94 (2.1%, 1.7–2.5)	65 (2.2%, 1.7–2.8)
K08 *	194 (2.6%, 2.3–3.0)	104 (2.3%, 1.9–2.8)	90 (3.1%, 2.5–3.8)
K09	7 (0.1%, 0.0–0.2)	6 (0.1%, 0.0–0.2)	1 (0.0%, 0.0–0.1)
K10	11 (0.1%, 0.1–0.2)	4 (0.1%, 0.0–0.2)	7 (0.2%, 0.1–0.4)
K11	11 (0.1%, 0.1–0.2)	6 (0.1%, 0.0–0.2)	5 (0.2%, 0.0–0.3)
K12	21 (0.3%, 0.2–0.4)	15 (0.3%, 0.2–0.5)	6 (0.2%, 0.1–0.4)
K13 **	41 (0.6%, 0.4–0.7)	16 (0.4%, 0.2–0.5)	25 (0.9%, 0.6–1.2)
K14	9 (0.1%, 0.1–0.2)	7 (0.2%, 0.1–0.3)	2 (0.1%, 0.0–0.2)

Source: Prepared by the authors using the results from the SIMEF database, January–December, 2022. 95% CI: 95% confidence interval. *n* = 7386. Females = 4496. Males = 2890. K00: Disorders of tooth development and eruption. K01: Embedded and impacted teeth. K02: Dental caries. K03: Other diseases of hard tissues of teeth. K04: Diseases of pulp and periapical tissues. K05: Gingivitis and periodontal diseases. K06: Other disorders of gingiva and edentulous alveolar ridge. K07: Dentofacial anomalies [including malocclusion]. K08: Other disorders of teeth and supporting structures. K09: Cysts of oral region, not elsewhere classified. K10: Other diseases of jaws. K11: Diseases of salivary glands. K12: Stomatitis and related lesions. K13: Other diseases of lip and oral mucosa. K14: Diseases of tongue. Comparisons between sex were performed using Yates’s continuity correction chi-square (* Yχ^2^ = 4.106, degree freedom = 1, *p* = 0.043; ** Yχ^2^ = 7.366, degree freedom = 1, *p* = 0.007).

**Table 3:** Age-stratified prevalence of oral diseases (ICD-10: K00–K14) among older adults according to the International Classification of Diseases, 10th Revision.

ICD-10 Codes	Sexagenarians *n*; % (95% CI)	Septuagenarians *n*; % (95% CI)	Octogenarians *n*; % (95% CI)	Nonagenarians *n*; % (95% CI)	Centenarians *n*; % (95% CI)
Total	3670	2406	1051	251	8
K00	2; 0.1% (0.0–0.1)	0; 0% (0.0–0.0)	1; 0.1% (0.0–0.3)	0; 0% (0.0–0.0)	0; 0% (0.0–0.0)
K01 *	12; 0.3% (0.2–0.5)	2; 0.1% (0.0–0.2)	0; 0% (0.0–0.0)	0; 0% (0.0–0.0)	0; 0% (0.0–0.0)
K02 *	83; 2.3% (1.8–2.8)	32; 1.3% (0.9–1.8)	7; 0.7% (0.2–1.1)	0; 0% (0.0–0.0)	0; 0% (0.0–0.0)
K03 *	26; 0.7% (0.4–1.0)	21; 0.9% (0.5–1.2)	1; 0.1% (0.0–0.3)	0; 0% (0.0–0.0)	0; 0% (0.0–0.0)
K04	60; 1.6% (1.2–2.1)	33; 1.4% (0.9–1.8)	8; 0.8% (0.3–1.3)	2; 0.8% (0.0–2.0)	0; 0% (0.0–0.0)
K05	39; 1.1% (0.7–1.4)	22; 0.9% (0.5–1.3)	3; 0.3% (0.0–0.7)	1; 0.4% (0.0–1.2)	0; 0% (0.0–0.0)
K06	14; 0.4% (0.2–0.6)	7; 0.3% (0.1–0.5)	0; 0% (0.0–0.0)	0; 0% (0.0–0.0)	0; 0% (0.0–0.0)
K07 *	97; 2.6% (2.1–3.2)	46; 1.9% (1.4–2.5)	14; 1.3% (0.7–2.0)	2; 0.8% (0.0–2.0)	0; 0% (0.0–0.0)
K08 *	116; 3.2% (2.6–3.7)	65; 2.7% (2.1–3.4)	11; 1.0% (0.5–1.7)	2; 0.8% (0.0–2.0)	0; 0% (0.0–0.0)
K09	6; 0.2% (0.1–0.3)	1; 0.0% (0.0–0.1)	0; 0% (0.0–0.0)	0; 0% (0.0–0.0)	0; 0% (0.0–0.0)
K10	7; 0.2% (0.1–0.4)	3; 0.1% (0.0–0.3)	1; 0.1% (0.0–0.3)	0; 0% (0.0–0.0)	0; 0% (0.0–0.0)
K11	3; 0.1% (0.0–0.2)	5; 0.2% (0.0–0.4)	3; 0.3% (0.0–0.7)	0; 0% (0.0–0.0)	0; 0% (0.0–0.0)
K12	10; 0.3% (0.1–0.5)	9; 0.4% (0.2–0.7)	2; 0.2% (0.0–0.5)	0; 0% (0.0–0.0)	0; 0% (0.0–0.0)
K13	23; 0.6% (0.4–0.9)	11; 0.5% (0.2–0.7)	7; 0.7% (0.2–1.2)	0; 0% (0.0–0.0)	0; 0% (0.0–0.0)
K14	4; 0.1% (0.0–0.2)	5; 0.2% (0.0–0.4)	0; 0% (0.0–0.0)	0; 0% (0.0–0.0)	0; 0% (0.0–0.0)

Source: Prepared by the authors using the results from the SIMEF database, January–December, 2022. 95% CI: 95% confidence interval. *n* = 7386. Females = 4496. Males = 2890. Sexagenarians (60–69 years old). Septuagenarians (70–79 years old). Octogenarians (80–89 years old). Nonagenarians (90–99 years old). Centenarians (≥100 years old). K00: Disorders of tooth development and eruption. K01: Embedded and impacted teeth. K02: Dental caries. K03: Other diseases of hard tissues of teeth. K04: Diseases of pulp and periapical tissues. K05: Gingivitis and periodontal diseases. K06: Other disorders of gingiva and edentulous alveolar ridge. K07: Dentofacial anomalies [including malocclusion]. K08: Other disorders of teeth and supporting structures. K09: Cysts of oral region, not elsewhere classified. K10: Other diseases of jaws. K11: Diseases of salivary glands. K12: Stomatitis and related lesions. K13: Other diseases of lip and oral mucosa. K14: Diseases of tongue. Comparisons between age group were performed using the likelihood ratio chi-square (LRχ^2^). * statistically significant *p* values. K00 (LRχ^2^ = 2.879, degree freedom [df] = 4, *p* = 0.578). * K01 (LRχ^2^ = 9.803, df = 4, *p* = 0.044). * K02 (LRχ^2^ = 25.917, df = 4, *p* < 0.001). * K03 (LRχ^2^ = 13.089, df = 4, *p* = 0.011). K04 (LRχ^2^ = 6.135, df = 4, *p* = 0.189). K05 (LRχ^2^ = 8.108, df = 4, *p* = 0.088). K06 (LRχ^2^ = 8.566, df = 4, *p* = 0.073). * K07 (LRχ^2^ = 11.706, df = 4, *p* = 0.020). * K08 (LRχ^2^ = 22.063, df = 4, *p* < 0.001). K09 (LRχ^2^ = 4.898, df = 4, *p* = 0.298). K10 (LRχ^2^ = 1.503, df = 4, *p* = 0.826). K11 (LRχ^2^ = 3.641, df = 4, *p* = 0.457). K12 (LRχ^2^ = 2.486, df = 4, *p* = 0.647). K13 (LRχ^2^ = 3.875, df = 4, *p* = 0.423). K14 (LRχ^2^ = 4.450, df = 4, *p* = 0.349).

**Table 4:** Sex-based distribution of the ten most frequent comorbidities in older adults with oral diseases (*n* = 384).

ICD-10 Codes	Total Population *n*; % (95% CI)	Females *n*; % (95% CI)	Males *n*; % (95% CI)
Total	384	229	155
1. I10.X	155; 40.4% (35.7–45.3)	97; 42.4% (36.0–48.8)	58; 37.4% (29.8–45.0)
2. E11.9	112; 29.2% (24.7–33.8)	69; 30.1% (24.2–36.1)	43; 27.7% (20.7–34.8)
3. E78	81; 21.1% (17.2–25.3)	53; 23.1% (17.7–28.6)	28; 18.1% (12.0–24.1)
E78.5	57; 14.8% (11.5–18.2)	37; 16.2% (11.4–20.9)	20; 12.9% (7.6–18.2)
E78.2	23; 6.0% (3.6–8.6)	16; 7.0% (3.7–10.3)	7; 4.5% (1.2–7.8)
E78.0	13; 3.4% (1.6–5.5)	8; 3.5% (1.1–5.9)	5; 3.2% (0.4–6.0)
E78.1	12; 3.1% (1.6–4.9)	8; 3.5% (1.1–5.9)	4; 2.6% (0.1–5.1)
4. E66	59; 15.4% (11.7–19.0)	38; 16.6% (11.8–21.4)	21; 13.5% (8.2–18.9)
5. I87.2	46; 12.0% (8.6–15.1)	33; 14.4% (9.9–19.0)	13; 8.4% (4.0–12.8)
6. J02.9	41; 10.7% (7.6–13.8)	21; 9.2% (5.4–12.9)	20; 12.9% (7.6–18.2)
7. E03.9 *	35; 9.1% (6.3–12.2)	30; 13.1% (8.7–17.5)	5; 3.2% (0.4–6.0)
8. M54.5 **	32; 8.3% (5.7–11.2)	25; 10.9% (6.9–15.0)	7; 4.5% (1.2–7.8)
9. N39.0 *	30; 7.8% (5.2–10.7)	26; 11.4% (7.2–15.5)	4; 2.6% (0.1–5.1)
10. F41.9 *	24; 6.3% (3.9–8.6)	22; 9.6% (5.8–13.4)	2; 1.3% (–0.5–3.1)

Source: Prepared by the authors using the results from the SIMEF database, January–December, 2022. 95% CI: 95% confidence interval. *N* = 384. Females = 229. Males = 155. I10.X: Essential (primary) hypertension. E11.9: Type 2 diabetes. E78: Chapter; disorders of lipoprotein metabolism and other lipidemias; E78.5: hyperlipidemia, unspecified; E78.2: mixed hyperlipidemia; E78.0: pure hypercholesterolemia; E78.1: pure hyperglyceridemia. E66: Chapter; obesity. I87.2: Venous insufficiency (chronic) (peripheral). J02.9: Acute pharyngitis. E03.9: Hypothyroidism. M54.5: Low back pain. N39.0: Urinary tract infection. F41.9: Anxiety disorder. Comparisons between sex were performed using Yates’s continuity correction chi-square (Yχ^2^) and Fisher’s exact test. * *p* value by Fisher’s exact test < 0.01. ** *p* value by Yχ^2^ = 0.042.

## Data Availability

The data presented in this study is not publicly available due to privacy and ethical restrictions. The dataset was obtained from the SIMEF and contains sensitive personal health information protected under data confidentiality regulations. Access to the data is therefore restricted to the principal investigators and authorized personnel only. Aggregated statistical data supporting the findings of this study are available from the corresponding authors upon reasonable request and subject to institutional approval.

## Supplementary Materials

The following supporting information can be downloaded at https://www.mdpi.com/article/10.3390/medicina62071325/s1, Table S1. Distribution of Recorded Diagnoses Across ICD-10 Chapters Among Older Adults in the Study Population (N = 2491 Diagnostic Records); Table S2. International Classification Diseases Tenth revision codes used to define oral and dental diseases (K00–K14) according to categories.. Figure S1: Flow Diagram of the Selection of the Study Population and Hierarchical Classification of ICD-10 Diagnostic Records for Oral Diseases Among Older Adults.

## Abbreviations

The following abbreviations are used in this manuscript:

95% CI	95% confidence interval
FMC	Family Medicine Clinic
ICD-10	International Classification of Diseases, Tenth Revision
IQR	Interquartile range
ISSSTE	Instituto de Seguridad y Servicios Sociales de los Trabajadores del Estado
LRχ^2^	Likelihood ratio chi-square
SD	Standard deviation
SIMEF	Medical Financial Information System
WHO	World Health Organization
Yχ^2^	Yates’s continuity correction chi-square
χ^2^	Chi-square test

## References

[1] Khan H.T.A., Addo K.M., Findlay H. (2024). Public Health Challenges and Responses to the Growing Ageing Populations. Public Health Chall..

[2] Noto S. (2023). Perspectives on Aging and Quality of Life. Healthcare.

[3] Hakeem F.F., Maharani A., Todd C., O’Neill T.W. (2023). Development, validation and performance of laboratory frailty indices: A scoping review. Arch. Gerontol. Geriatr..

[4] Aïdoud A., Gana W., Poitau F., Debacq C., Leroy V., Nkodo J.A., Poupin P., Angoulvant D., Fougère B. (2023). High Prevalence of Geriatric Conditions Among Older Adults With Cardiovascular Disease. J. Am. Heart Assoc..

[5] Lopez-Hernandez D., Brito-Aranda L., Liceaga-Perez L.G., Beltran-Lagunes L., Hernandez-Ramirez G.E., Thompson-Bonilla M.R., Castro-Diaz A.M., León-Jiménez R.G., Vazquez-Sanchez A., Melgarejo-Estefan E. (2025). Epidemiological Association between Oral Diseases and COVID-19: Pulpitis as a Key Risk Factor. Curr. J. Appl. Sci. Technol..

[6] Lopez-Hernandez D., Brito-Aranda L., Liceaga-Perez L.G., Vazquez-Sanchez A., Lopez-Sanchez M.A., Ramirez-Garcia A., Torres-Garcia E.E., Jimenez-Hernandez R.L., Saldivar-Gonzalez M.L.L., Salinas-Palacios P.V. (2025). Clinical and Sociodemographic Characteristics of Patients with Diabetes in Primary Care Services. Curr. J. Appl. Sci. Technol..

[7] Lopez Hernandez D., Brito Aranda L., Liceaga Perez L.G., Melgarejo Estefan E., Vazquez Sanchez A., Olivares Lopez X.L., Noguez Alvarez V.H., Saldivar Gonzalez M.L.L., Carrera Vite A.V., Hernandez Almazan M.C. (2025). Epidemiological Profile of Comorbidities and Communicable Diseases, and Age- and Sex-Specific Distribution in Mexican Adults with Dyslipidaemia Attended in Primary Care Services: A Cross-Sectional Analysis. Biomed. J. Sci. Technol. Res..

[8] Lopez Hernandez D., Brito Aranda L., Meneses Mayo M., Cruz Aviles E., Hernandez Almazan M.C., Vazquez Sanchez A., Torres Garcia E.E., Ramirez Velazquez C., Saldivar Gonzalez M.L.L., Jimenez Hernandez R.L. (2026). Comorbidities and Communicable Diseases in Mexican Adults with Hypertension in Primary Care: An Age- and Sex-Stratified Cross-Sectional Study. Biomed. J. Sci. Technol. Res..

[9] Fabbri E., Candia J., Tanaka T., Moore A.Z., Muratori P., Brugiavini A., Calderón-Larrañaga A., Vetrano D.L., Fratiglioni L., Crimmins E. (2025). Dynamics of multimorbidity, health expectancy, and survival in middle aged and older individuals. J. Gerontol. Ser. A Biol. Sci. Med. Sci..

[10] Aggarwal P., Woolford S.J., Patel H.P. (2020). Multi-Morbidity and Polypharmacy in Older People: Challenges and Opportunities for Clinical Practice. Geriatrics.

[11] Wu D., Xu J., Zhang H., Zhang K., Zhu Y. (2025). Multimorbidity characteristics in older adults and their associated factors in complex networks: A cross-sectional study. Front. Public Health.

[12] Carrera-Vite A.V., Lopez-Hernandez D., Brito-Aranda L., Liceaga-Perez L.G., Hernandez-Almazan M.C., Cruz-Aviles E., Saldivar-Gonzalez M.L.L., Vazquez-Sanchez A., Olivares-Lopez X.L., Noguez-Alvarez V.H. (2026). Epidemiological Profile and Healthcare Utilization Patterns in a Family Medicine Clinic in Northern Mexico City: A Stratified Analysis by Sex and Age. Biomed. J. Sci. Tech. Res..

[13] Lopez-Hernandez D., Brito-Aranda L., Flores-Morales G.J., Ham-Olvera M.C., Beltran-Lagunes L., Vazquez-Sanchez A., Jimenez-Hernandez R.L., Melgarejo-Estefan E., Torres-García E.E., Olivares-Lopez X.L. (2024). Health status and demographic characteristics of patients attending a primary care unit in Mexico City: A descriptive study. Curr. J. Appl. Sci. Technol.

[14] Osterholm M.T., Hedberg C.W. (2015). Epidemiologic Principles. Mandell, Douglas, and Bennett’s Principles and Practice of Infectious Diseases.

[15] Detels R. (2015). Epidemiology: The foundation of public health. Oxford Textbook of Global Public Health.

[16] Krämer A., Akmatov M., Kretzschmar M. (2009). Principles of Infectious Disease Epidemiology. Modern Infectious Disease Epidemiology: Concepts, Methods, Mathematical Models, and Public Health.

[17] Kaslow R.A. (2014). Epidemiology and Control: Principles, Practice and Programs. Viral Infections of Humans: Epidemiology and Control.

[18] Huang X., Kang L., Bi J. (2025). Epidemiology of oral health in older adults aged 65 or over: Prevalence, risk factors and prevention. Aging Clin. Exp. Res..

[19] Shan W., Feng Q., Yao L., Cen M., Zhou X., Ma L., Chen J., Zhou W., Chen C., Chen M. (2026). Burden of Oral Diseases in Adults Aged 70 Years and Older: Time-Trend Analysis of Global Burden of Disease Study 2021. Oral Dis..

[20] Dibello V., Zupo R., Sardone R., Lozupone M., Castellana F., Dibello A., Daniele A., De Pergola G., Bortone I., Lampignano L. (2021). Oral frailty and its determinants in older age: A systematic review. Lancet. Healthy Longev..

[21] Chan A.K.Y., Chu C.H., Ogawa H., Lai E.H. (2024). Improving oral health of older adults for healthy ageing. J. Dent. Sci..

[22] Vizcaíno K., Armas A. (2022). Prevalencia de edentulismo en adultos mayores en América Latina. Revisión de literatura. Rev. Estomatológica Hered..

[23] Beck J.D., Youngblood M., Atkinson J.C., Mauriello S., Kaste L.M., Badner V.M., Beaver S., Becerra K., Singer R. (2014). The prevalence of caries and tooth loss among participants in the Hispanic Community Health Study/Study of Latinos. J. Am. Dent. Assoc..

[24] Jiang H., Yin L., Hu Z., Chen Z., Yue H., Qin Z. (2025). Global, regional, and national temporal trends of edentulism burden from 1990 to 2021 and predictions to 2050: An age-period-cohort analysis, decomposition analysis and frontier analysis. J. Stomatol. Oral Maxillofac. Surg..

[25] Wei T., Du Y., Hou T., Zhai C., Li Y., Xiao W., Liu K. (2023). Association between adverse oral conditions and cognitive impairment: A literature review. Front. Public Health.

[26] Thu Ya M., Hasegawa Y., Sta Maria M.T., Hattori H., Kusunoki H., Nagai K., Tamaki K., Hori K., Kishimoto H., Shinmura K. (2024). Predicting cognitive function changes from oral health status: A longitudinal cohort study. Sci. Rep..

[27] Chen J.T., Tsai S., Chen M.H., Pitiphat W., Matangkasombut O., Chiou J.M., Han M.L., Chen J.H., Chen Y.C. (2024). Association between oral health and cognitive impairment in older adults: Insights from a Six-year prospective cohort study. J. Dent..

[28] Xie Y., Xia X., Tian X., Hu Y., Li Y., Tan X., Wu W., Dong B., Wang Y. (2025). The association between cognitive impairment and oral health or oral hygiene behaviors among multiethnic older adults in Western China: A cross-sectional multicenter study. BMC Public Health.

[29] Mishima Y., Nakamura M., Matsuda Y., Nishi K., Takaoka R., Kanno T., Takenaka T., Tabira T., Makizako H., Kubozono T. (2025). Association Between Cognitive Impairment and Poor Oral Function in Community-Dwelling Older People: A Cross-Sectional Study. Healthcare.

[30] Ye N., Deng B., Hu H., Ai Y., Liu X., Zhou S., Li Y. (2024). The association between oral health and mild cognitive impairment in community-dwelling older adults. Front. Public Health.

[31] Lipsky M.S., Singh T., Zakeri G., Hung M. (2024). Oral Health and Older Adults: A Narrative Review. Dent. J..

[32] Vaishampayan P., Beniwal J.S., Wilk P., McLean S., Jessani A. (2025). Unmet oral health needs and barriers to dental services among socially marginalized youth: A scoping review. Front. Oral Health.

[33] de Abreu M.H.N.G., Cruz A.J.S., Borges-Oliveira A.C., Martins R.d.C., Mattos F.d.F. (2021). Perspectives on Social and Environmental Determinants of Oral Health. Int. J. Environ. Res. Public Health.

[34] Janto M., Iurcov R., Daina C.M., Neculoiu D.C., Venter A.C., Badau D., Cotovanu A., Negrau M., Suteu C.L., Sabau M. (2022). Oral Health among Elderly, Impact on Life Quality, Access of Elderly Patients to Oral Health Services and Methods to Improve Oral Health: A Narrative Review. J. Pers. Med..

[35] Korczeniewska O.A., Eliav E., Arany S. (2025). Medication-Induced Xerostomia: Cross-Sectional Analysis of Salivary Flow, Intraoral Aching, and Anxiety. J. Clin. Med..

[36] Wolff A., Joshi R.K., Ekström J., Aframian D., Pedersen A.M., Proctor G., Narayana N., Villa A., Sia Y.W., Aliko A. (2017). A Guide to Medications Inducing Salivary Gland Dysfunction, Xerostomia, and Subjective Sialorrhea: A Systematic Review Sponsored by the World Workshop on Oral Medicine VI. Drugs RD.

[37] Ji H., Su S., Chen M., Liu S., Liu S., Guo J. (2025). The role of gut microbiota in insulin resistance: Recent progress. Front. Microbiol..

[38] Caricilli A.M., Saad M.J.A. (2013). The Role of Gut Microbiota on Insulin Resistance. Nutrients.

[39] Celis A., Cáceres B., Escobar B., Barahona P., Dreyer E., Petermann-Rocha F. (2025). Impact of Oral Health Interventions on Sarcopenia and Frailty in Older Adults: A Systematic Review. J. Clin. Med..

[40] Sánchez-Sánchez J.L., Lu W.H., Gallardo-Gómez D., Del Pozo Cruz B., de Souto Barreto P., Lucia A., Valenzuela P.L. (2024). Association of intrinsic capacity with functional decline and mortality in older adults: A systematic review and meta-analysis of longitudinal studies. Lancet. Healthy Longev..

[41] Belloni G., Cesari M. (2019). Frailty and Intrinsic Capacity: Two Distinct but Related Constructs. Front. Med..

[42] Grigoraș G., Ilie A.C., Turcu A.-M., Albișteanu S.-M., Lungu I.-D., Ștefăniu R., Pîslaru A.I., Gavrilovici O., Alexa I.D. (2025). Resilience and Intrinsic Capacity in Older Adults: A Review of Recent Literature. J. Clin. Med..

