# Supplementary material for: Burden and Patterns of Oral Diseases and Systemic Comorbidities in Older Adults Attending Primary Care: A Sex- and Age-Stratified Analysis
Source: Medicina (Kaunas). 2026 Jul 9;62(7):1325. doi: 10.3390/medicina62071325 (PMC13413689; doi:10.3390/medicina62071325)
Supplement: Supplementary file 1 [file medicina-62-01325-s001.zip › Supplement Figure S1.pdf]

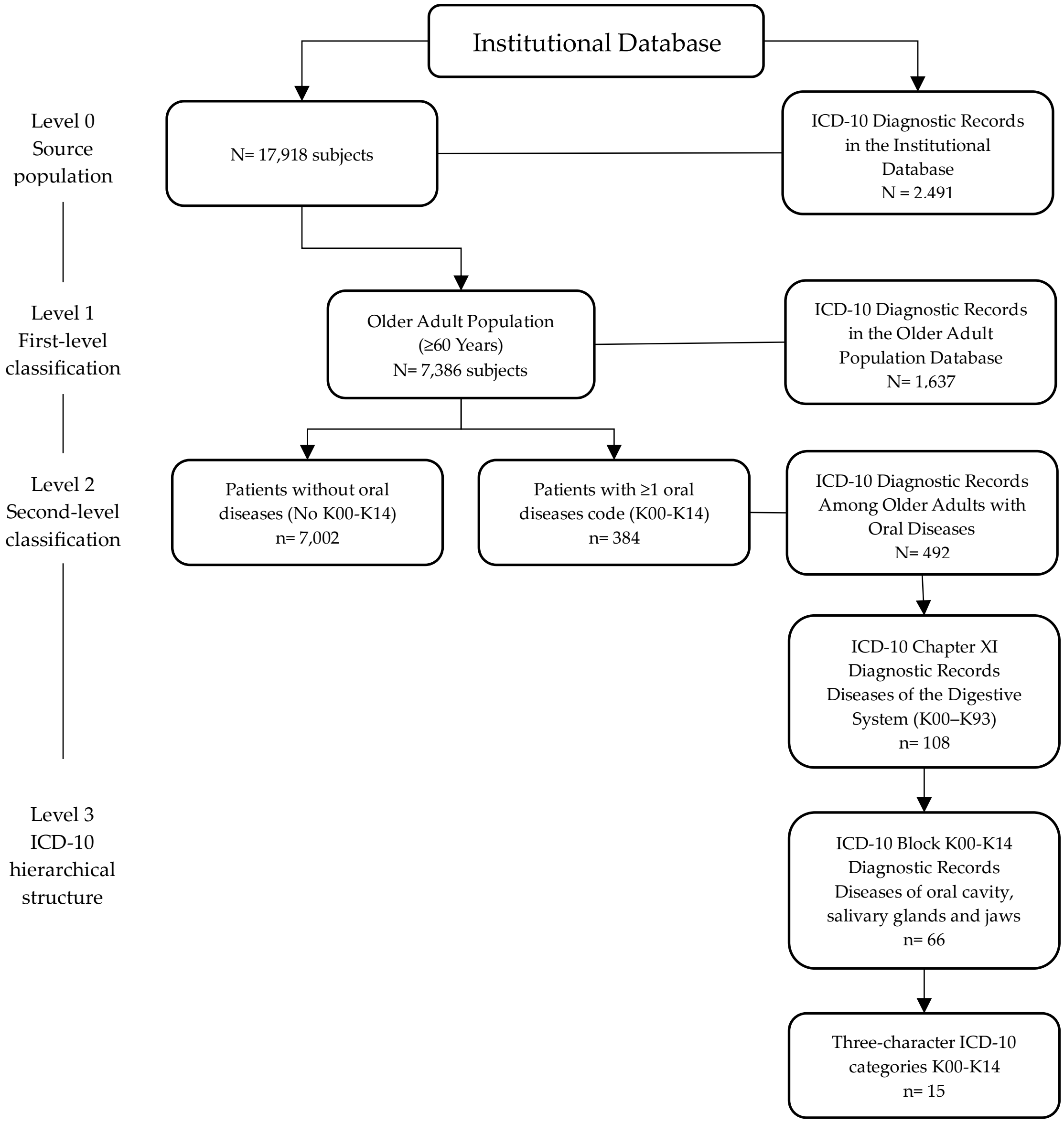

Figure S1. Flow Diagram of the Selection of the Study Population and Hierarchical Classification of ICD-10 Diagnostic Records for Oral Diseases Among Older Adults.
