# Supplementary material for: Burden and Patterns of Oral Diseases and Systemic Comorbidities in Older Adults Attending Primary Care: A Sex- and Age-Stratified Analysis
Source: Medicina (Kaunas). 2026 Jul 9;62(7):1325. doi: 10.3390/medicina62071325 (PMC13413689; doi:10.3390/medicina62071325)
Supplement: Supplementary file 1 [file medicina-62-01325-s001.zip › Supplementary Tables S1 and S2.pdf]

Table S1. Distribution of Recorded Diagnoses Across ICD-10 Chapters Among Older Adults in the Study Population (N = 2,491 Diagnostic Records).

**ICD-10 Chapter**	**Description**	**n (%)**
I	Certain infectious and parasitic diseases (A00–B99)	127 (5.1)
II	Neoplasms (C00–D48)	170 (6.8)
III	Diseases of the blood and blood-forming organs and certain disorders involving the immune mechanism (D50–D89)	39 (1.6)
IV	Endocrine, nutritional and metabolic diseases (E00–E90)	113 (4.5)
V	Mental and behavioural disorders (F00–F99)	151 (6.1)
VI	Diseases of the nervous system (G00–G99)	123 (4.9)
VII	Diseases of the eye and adnexa (H00–H59)	120 (4.8)
VIII	Diseases of the ear and mastoid process (H60–H95)	46 (1.8)
IX	Diseases of the circulatory system (I00–I99)	157 (6.3)
X	Diseases of the respiratory system (J00–J99)	93 (3.7)
XI	Diseases of the digestive system (K00–K93)	223 (9.0)
XII	Diseases of the skin and subcutaneous tissue (L00–L99)	118 (4.7)
XIII	Diseases of the musculoskeletal system and connective tissue (M00–M99)	239 (9.6)
XIV	Diseases of the genitourinary system (N00–N99)	164 (6.6)
XV	Pregnancy, childbirth and the puerperium (O00–O99)	28 (1.1)
XVI	Certain conditions originating in the perinatal period (P00–P96)	7 (0.3)
XVII	Congenital malformations, deformations and chromosomal abnormalities (Q00–Q99)	79 (3.2)
XVIII	Symptoms, signs and abnormal clinical and laboratory findings, not elsewhere classified (R00–R99)	106 (4.3)
XIX	Injury, poisoning and certain other consequences of external causes (S00–T98)	250 (10.0)
XX	External causes of morbidity and mortality (V01–Y98)	16 (0.6)
XXI	Factors influencing health status and contact with health services (Z00–Z99)	116 (4.7)
XXII	Codes for special purposes (U00–U99)	6 (0.2)
Total		2,491

Source: Prepared by the authors using the results from the SIMEF database, January-December, 2022. Percentages were calculated using the total number of ICD-10 diagnostic codes recorded in the institutional database (N = 2,491).

Table S2. International Classification Diseases Tenth revision codes used to define oral and dental diseases (K00–K14) according to categories.

**Subcategory (ICD-10)**	**Codes included**	**Description**
K00	K00.0; K00.2	Disorders of tooth development and eruption
K01	K01.0; K01.1; K01.2	Embedded and impacted teeth
K02	K02.0; K02.1; K02.3; K02.9	Dental caries
K03	K03.0; K03.1; K03.2; K03.3; K03.6; K03.8; K03.9	Other diseases of hard tissues of teeth
K04	K04.0; K04.1; K04.2; K04.3; K04.4; K04.5; K04.6; K04.7; K04.8; K04.9	Diseases of pulp and periapical tissues
K05	K05.0; K05.1; K05.2; K05.3; K05.4; K05.6	Gingivitis and periodontal diseases
K06	K06.0; K06.2; K06.3; K06.8; K06.9	Other disorders of gingiva and edentulous alveolar ridge
K07	K07.0; K07.2; K07.5; K07.6; K07.8; K07.9	Dentofacial anomalies (including malocclusion)
K08	K08.1; K08.3; K08.8; K08.9	Other disorders of teeth and supporting structures
K09	K09.0; K09.2; K09.8; K09.9	Cysts of oral región
K10	K10.0; K10.1; K10.3; K10.8	Other diseases of jaws
K11	K11.2; K11.7; K11.8	Diseases of salivary glands
K12	K12.0; K12.1; K12.2	Stomatitis and related lesions
K13	K13.0; K13.2; K13.7	Diseases of lip and oral mucosa
K14	K14.8; K14.9	Diseases of tongue

Source: Prepared by the authors using the results from the SIMEF database, January-December, 2022.
